# Fabrication of Mechanically Robust Hydrophobic Surfaces Using Femtosecond Laser Shock Peening

**DOI:** 10.3390/ma18092154

**Published:** 2025-05-07

**Authors:** Chao Xu, Mengyu Jia, Yucheng Gu, Peishuo Wang, Zhen Zhang, Yulei Wang

**Affiliations:** 1 AECC Beijing Institute of Aeronautical Materials, Beijing 100095, China; 2 Center for Advanced Laser Technology, School of Electronics and Information Engineering, Hebei University of Technology, Tianjin 300401, China; 3 Hebei Key Laboratory of Advanced Laser Technology and Equipment, Tianjin 300401, China

**Keywords:** femtosecond laser shock peening, hydrophobic, mechanical properties

## Abstract

The harsh service environment has increased the demand for hydrophobic surfaces with excellent mechanical properties; however, how to manufacture such surfaces remains a significant challenge. In this study, a method for fabricating hydrophobic surfaces with excellent mechanical properties using femtosecond laser shock peening (fs-LSP) is proposed, without the need for any additional processing steps. Taking CH1900A martensitic steel as an example, a systematic analysis of the microstructure was conducted after fs-LSP, revealing the mechanisms by which fs-LSP affects surface morphology, grain structure, dislocation density, and grain boundary characteristics. The high-density dislocations and grain refinement induced by fs-LSP significantly enhanced the surface hardness and introduced residual compressive stresses. Additionally, the laser-induced periodic micro/nanostructures on the surface ensured excellent hydrophobic properties. The effect of single pulse energy and the number of impacts on fs-LSP has also been discussed in detail. As the pulse energy and number of impacts were increased, the surface microstructure of the material was progressively optimized, evidenced by grain refinement, an increase in geometrically necessary dislocation (GND) density, and a higher proportion of high-angle grain boundaries (HAGBs). Such optimization is not monotonous or unlimited; a pulse energy of 75 μJ and six impacts achieved the optimal effect, with the surface hardness reaching up to 8.2 GPa and a contact angle of 135 degrees. The proposed fs-LSP provides a new strategy for manufacturing hydrophobic surfaces with excellent mechanical properties, and the detailed discussion and analysis also provide theoretical guidance for process optimization.

## 1. Introduction

Hydrophobic surfaces are essential in a wide range of applications, including self-cleaning [1,2], oil–water separation [3], anti-icing [4], and anti-corrosion [5]. In response to the increasingly harsh service environments characterized by various loads, such as cyclic mechanical loading and abrasion, hydrophobic surfaces with mechanical durability have sparked significant research interest [6]. Although various methods, such as chemical etching [7] and laser texturing [8], have successfully manufactured hydrophobic surfaces, rapid failure under continuous loading poses a threat to their service performance due to weak mechanical properties. Typical mechanical properties mainly include surface hardness, wear resistance, strength-plasticity, and fatigue performance of materials. A common way for the degradation in hydrophobic performance to occur is that the hydrophobic structure is damaged or even directly removed under wear loads because the surface is too soft. In addition, if the strength–plasticity or fatigue performance is too low, the component may directly fracture under tensile or cyclic loads. Therefore, it is crucial to improve the mechanical properties of the hydrophobic surface to resist the degradation of wettability or material failure. To date, the development of hydrophobic surfaces with excellent mechanical properties remains an open field.

The meticulous design of micro/nanostructures was initially employed to enhance the mechanical properties of hydrophobic surfaces. Chen et al. fabricated hydrophobic, anti-icing surfaces enriched with CuO nanowires by employing laser texturing followed by additional heat treatment [9]. The pre-fabricated micro/nanostructures on the surface released thermal stress, significantly enhancing the adhesion strength of the nanowires. After ten tape tests, the CuO nanowire surface still maintained its super-hydrophobicity. Unfortunately, the complex process flow and uneven growth of nanowires pose significant challenges for large-scale industrial application. Deng et al. proposed a solution that entails the fabrication of pyramid-like armor structures on metal surfaces via mechanical high-pressure deformation [10]. This method effectively preserves the integrity of the internal inverted pyramid structure even under intense external mechanical loads, thereby achieving a significant hydrophobic effect. Notably, the micro-pyramid array requires the photolithography technology to pre-fabricate molds, which is both time-consuming and costly. Additionally, the pyramid-shaped tip structures are often stress concentration points, making them prone to crack initiation and eventual failure during service. Therefore, it is imperative to continue exploring ideal manufacturing methods for hydrophobic surfaces with superior mechanical properties.

Laser shock peening (LSP) can enhance the surface mechanical properties of materials while introducing controlled micro/nanostructures, making it a potential solution for manufacturing hydrophobic surfaces with mechanical durability [11,12,13]. LSP induces plasma shock waves on the target material surface, leading to plastic deformation, microstructural transformation, and residual stresses, thereby significantly enhancing mechanical properties such as fatigue and wear resistance [14,15,16]. Moreover, hydrophobic surfaces with arbitrary micro/nanostructures can be manufactured by designing laser scanning paths. Zhang et al. proposed a 3D-gradient nanosecond laser shock peening method, which creates multi-scale gradient structures on the material surface to ultimately form a self-armored hydrophobic surface [6]. While maintaining excellent hydrophobic properties, the yield strength, uniform elongation, and other mechanical properties of the material were significantly enhanced. In another study, the 3D gradient nanosecond laser shock peening method was further combined with heating, creating hydrophobic surfaces on 7075 aluminum alloy and improving the material’s bending fatigue performance [17]. A key factor of the aforementioned method is laying a thin metal mesh on the substrate surface and securing it with a matching fixture. Additionally, previous shock peening processes have predominantly utilized nanosecond lasers, necessitating the use of a confinement layer (typically water) and an absorption layer (typically tape or aluminum foil) to enhance the shock-hardening effect [18]. Therefore, the applicability of these methods to components with complex surfaces poses a significant challenge, for example, gear roots are difficult to uniformly cover with metal mesh, confinement layers, and absorption layers. Moreover, a typical drawback of nanosecond laser shock peening is the significant surface roughness it induces on the material, which greatly compromises mechanical properties, especially fatigue resistance [19]. Femtosecond lasers, owing to their extremely high fluence and processing precision, can achieve shock peening without confinement and absorption layers, thus allowing for versatile applications in various complex scenarios [20]. Chen et al. successfully implemented the femtosecond laser shock peening (fs-LSP) of commercially pure copper without a confinement layer and absorptive layer, achieving a surface roughness approximately 1/9 to 1/7 that of nanosecond laser [21]. Additionally, femtosecond lasers are also an effective method for fabricating hydrophobic micro/nanostructures on material surfaces. Kumthekar et al. utilized femtosecond laser to fabricate hydrophobic micro/nanostructures on the surface of Ni-Mn-Ga-based alloys, demonstrating that the wettability can be flexibly controlled by adjusting the laser processing parameters [22]. Therefore, developing fs-LSP processes and strategies is an ideal solution for manufacturing functional surfaces that combine mechanical performance and hydrophobic properties. However, systematic research and analysis on the enhancement of surface mechanical properties and wettability, as well as the underlying mechanisms, following fs-LSP processes, are nearly absent.

In this study, a method for fabricating hydrophobic surfaces possessing outstanding mechanical properties through fs-LSP is introduced, eliminating the necessity for a confinement layer, absorption layer, or further processing stages. Taking the fourth-generation high-strength gear steel CH1900A as an example, fs-LSP successfully enhanced its mechanical properties and hydrophobic performance. A systematic analysis of the surface micro/nanostructures and subsurface microstructural transformation induced by femtosecond laser revealed the mechanisms underlying the performance enhancements. Furthermore, the effects of pulse energy and the number of impacts on the fs-LSP process were discussed in detail. The proposed method and research results provide a theoretical foundation and process guidance for manufacturing advanced hydrophobic surfaces with excellent mechanical properties.

## 2. Experimental Procedure

### 2.1. Materials and fs-LSP Processing

The target material used is the fourth-generation high-strength gear steel CH1900A, which is widely employed in critical components such as gears in aerospace engines. The material was cut into thin sheets measuring 10 mm × 10 mm × 2 mm. Before fs-LSP treatment, the sample surfaces were polished, followed by 20 min of ultrasonic cleaning in alcohol to remove any contaminants. The sample was first ground with 400#–2000# grit sandpaper. Subsequently, the polishing process was conducted on a mechanical polishing machine at a rotational speed of 600 r/min, with a diamond abrasive being employed as the polishing fluid. The polished specimens exhibited surface roughness in the range of 10–20 nm.

**Figure 1:**
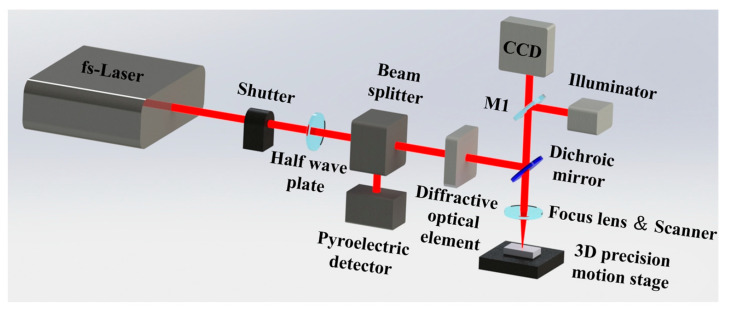
The diagram of the experimental setup for fs-LSP process.

A commercial femtosecond laser (Light Conversion, Vilnius, Lithuania) was employed for fs-LSP processing, generating 260 fs pulses at a wavelength of 1030 nm. The frequency was fixed at 1 kHz, and the laser beam exhibited a Gaussian spatial distribution with a spot diameter of 50 μm. The laser beam diameter was measured using the knife-edge method. The laser beam was first split into two paths, with a half-wave plate regulating the energy of the secondary pulse. Subsequently, the low-energy beam was directed to an energy meter while a precision blade progressively occluded the laser beam. The positions where the detected energy reached 10% and 90% of the total were recorded. The beam radius was calculated by subtracting these two positions and multiplying the result by 0.78, based on the Gaussian beam propagation model. A half-wave plate and beam splitter assembly was employed to control the input energy. Real-time monitoring of laser power was achieved using a pyroelectric detector in conjunction with a beam splitter. The proportion of transmitted light can be adjusted by rotating the half-wave plate, thereby regulating the output energy. The beam is subsequently split into two components via a polarizing beam splitter (PBS), accounting for 1% and 99% of the total energy, respectively. A total of 99% of the energy is utilized for shock peening on the target surface, whereas 1% of the energy is vertically incident on a pyroelectric detector to monitor the laser power. The total laser power can be calculated based on the energy ratio between these two components. Here, the effects of pulse energy and the number of impacts on fs-LSP are the focal points of this research. A total of 7 groups of experiments were conducted. When the number of impacts was fixed at 1, four groups of different single pulse energies were set, which were 25 μJ, 50 μJ, 75 μJ, and 100 μJ, respectively. Similarly, when the single pulse energy was fixed at 75 μJ, four groups of different numbers of impacts were set, which were 1, 3, 6, and 9, respectively. During fs-LSP, the sample was fixed on a five-axis displacement platform, with the laser beam directly focused onto the sample surface. For each set of laser parameters, the laser scanned in a zig-zag pattern to form a square area of 5 mm × 5 mm. The scanning speed was 20 mm/s, and the overlap rate was set to 50%. Multiple laser impacts on the surface of the target material are achieved by repeating the same process N times. Figure 1 shows the diagram of the experimental setup for the fs-LSP process.

### 2.2. Microstructure Characterization and Mechanical Property Testing

After fs-LSP, the surface roughness was first characterized by laser scanning confocal microscopy (LSCM, LEXT OLS5100, Olympus, Tokyo, Japan). A linear scan was performed along the direction perpendicular to the laser-induced periodic surface structures (LIPSSs) to obtain the surface geometric profile. The Ra could be calculated based on the amplitude of the profile line fluctuations within a certain length range. For each set of fs-LSP parameters, the tests were repeated three times, and the average value was taken as the final Ra. Subsequently, specimens in cross-section cut from the top surface along the laser incidence direction were prepared. The specimens were initially ground through successive stages using sandpaper up to 2000-grit, followed by mechanical polishing with diamond abrasive paste until achieving a mirror-like surface with a final roughness of approximately 10–20 nm. After etching the cross-section with a 4% nital solution, the surface morphology was characterized by scanning electron microscopy (SEM, ZEISS Sigma300, Jena, Germany). Furthermore, electron backscatter diffraction (EBSD) was employed to characterize the grain information of the cross-section samples. Before EBSD testing, the samples were subjected to vibratory polishing for approximately 8 h.

The performance tests of the material after fs-LSP include two aspects: hardness and wetting properties. Regarding the hardness, the nanoindentation tests were performed using a T1 Premier system manufactured by Bruker (Bremen, Germany). The indenter type was a 3-sided pyramid. The microhardness values were measured at 2 μm intervals from the surface to the interior of the sample cross-section until no significant changes were observed to evaluate the affected thickness of fs-LSP. For each depth, the test was repeated 5 times, and the average value was taken as the final hardness. The residual stress is further calculated from the hardness obtained by nano-indentation, following the method proposed by Suresh and Giannakopoulos, as follows [23]:

(1) $$\frac{A_{0}}{A}=\left\{\begin{array}{c} 1+\frac{\sigma_{R}}{H}\;\;\;\;\;\;\;\;\;\;\;\;\;\;\sigma_{R}>0\\ 1+\frac{\sigma_{R}sin\alpha }{H}\;\;\;\;\;\;\sigma_{R}<0 \end{array}\right.$$

where *A*_0_ is the contact area of the sample without residual stress; *A* is the contact area of the sample with residual stress; *σ_R_* is the internal residual compressive stress of the sample; *H* is the hardness; and *α* is the complementary angle of the half-included angle of the indenter tip. In this paper, a diamond Berkovich indenter tip was used, and the value of α is 24.7°.

The wettability test is carried out immediately within 1–2 h after fs-LSP. A CCD camera was used to capture a transverse snapshot of the droplets in the area, and the droplet contact angle (CA) was measured by the ImageJ 2024 plug-in Contact Angle. The CCD camera was interfaced with a varifocal lens while maintaining precise height alignment with the specimen. An LED light source was positioned on the opposite side of the sample to provide trans-illumination. The CCD imaging system (XDC-10A, Hayear, Jiaxing, China) was utilized for optical acquisition. In the CA tests, distilled water droplets with a volume of 2 µL were utilized. All the CA results are the averages of 5 measurements.

## 3. Results

### 3.1. Surface Morphology and Roughness

**Figure 2:**
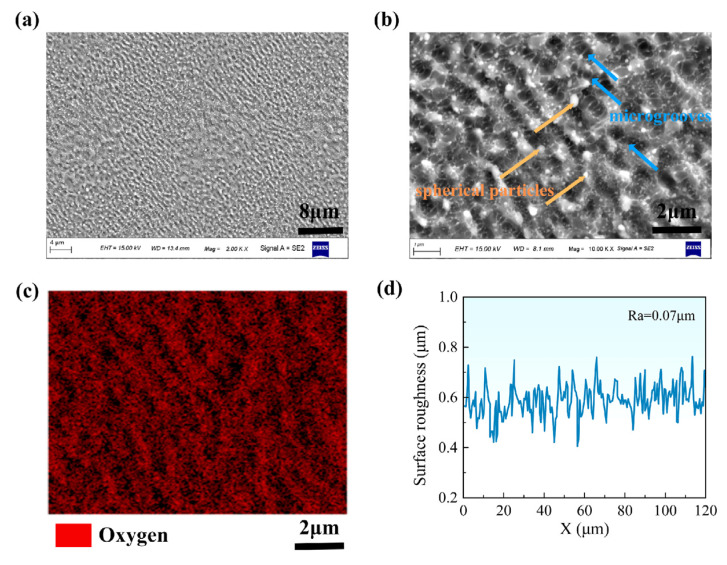
Surface morphology and roughness after fs-LSP. (**a**,**b**) Surface morphology of periodic micro/nanostructures; (**c**) distribution of oxygen elements corresponding to (**b**); (**d**) surface geometric contour.

The surface morphology and roughness of the material after fs-LSP treatment are first characterized, which are the key factors affecting the wettability and mechanical properties. After a single fs-LSP treatment with a pulse energy of 75 μJ, as shown in Figure 2a,b, a non-uniform micro-nano dual-scale structure formed on the sample surface, including micron-scale LIPSSs and spherical particles with diameters of several nanometers. Additionally, numerous microgrooves are present between the LIPSSs, which are attributed to the combined effect of material removal caused by laser ablation and slight plastic deformation caused by the shock wave. The formation of the LIPSSs is attributed to the redistribution of incident laser by surface plasmon polaritons, which occurs during the ablation stage [24,25]. The nanoscale particles are the result of splashing and re-deposition. Figure 2c shows the compositional distribution of surface micro/nanostructures obtained by EDS, highlighting the oxygen content of the nanoparticles and LIPSSs is significantly higher than that of the substrate. During fs-LSP, partial target materials are instantaneously excited into plasma, forming a continuous ablative plume that includes numerous liquid clusters detached from the substrate. Due to the absence of confinement and absorber layers, the liquid clusters oxidize rapidly in air and re-deposit onto the peening surface. Previous studies have shown that the LIPSSs and oxides produced by femtosecond laser ablation may hinder surface hardening. However, the dual-scale micro/nanostructures are advantageous for enhancing surface hydrophobicity. It is ideal to seek a balance between the conflicting requirements of mechanical properties and wettability for LIPSSs and oxide nanoparticles by controlling the laser processing parameters. The effects of fs-LSP parameters will be discussed in detail in Section 4. Figure 2d shows the 3D morphology of the surface micro/nanostructures. After fs-LSP, the surface roughness Ra is only 0.07 μm, with LIPSSs exhibiting a characteristic period of approximately 800 nm. In contrast, for nanosecond laser shock peening, this value typically exceeds 2 μm due to more pronounced thermal effects [26].

### 3.2. Cross-Section Microstructure

**Figure 3:**
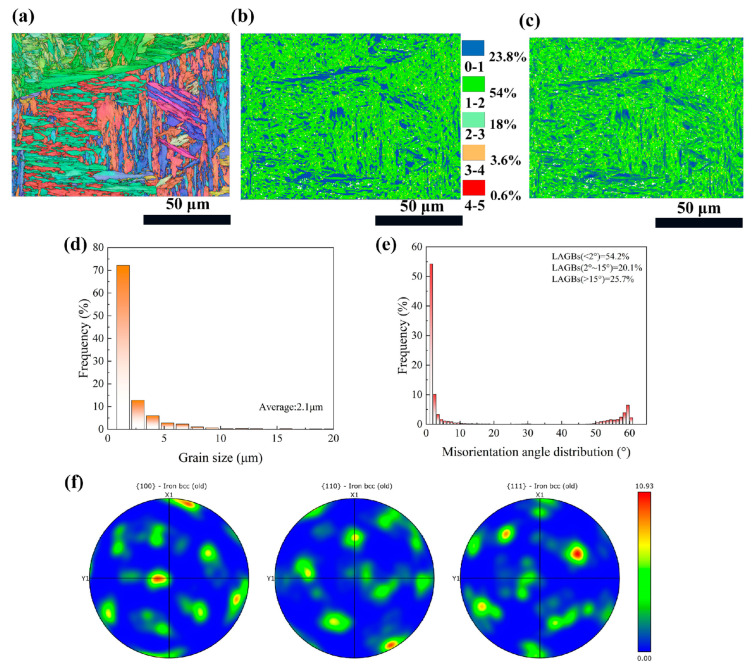
Cross-sectional EBSD results of as-received sample. (**a**) IPF image; (**b**) KAM image; (**c**) GND image; (**d**) grain size distribution; (**e**) misorientation angle distribution; (**f**) PF image.

Cross-sectional EBSD images of the as-received sample are displayed in Figure 3. To facilitate comparison with subsequent fs-LSP samples, the EBSD testing region was positioned adjacent to the upper surface of the sample. As shown in the IPF map, the CH1900A matrix consists of a substantial amount of randomly textured lath martensite, within which numerous fine equiaxed grains are present. The original austenite boundaries segment the martensite growth regions, and within each region, a slight preferential orientation is observed. The grain sizes are predominantly distributed in the range of 1–2.5 μm (Figure 3d), with an average size of 2.1 μm. Figure 3b is the kernel average misorientation (KAM) map, which displays the distribution of the average misorientation between the data point and all of its neighbors. The majority of the KAM values are relatively low, with over 77.8% of the misorientations being less than 2 degrees. The distribution of KAM values is generally considered to represent, to some extent, the distribution of dislocation density. The distribution of local geometrically necessary dislocations (GND), calculate based on KAM values, show that higher GND density coincided with regions of higher KAM values. The maximum GND density is approximately 31.1 × 10^14^/m^2^. The misorientation angle distributions are further analyzed (Figure 3e), revealing that 74.3% of the misorientation angles in the as-received sample are less than 15 degrees. The as-received sample underwent a tempering recrystallization process, forming new grains with low dislocation density and low residual stress within the material. These new grains exhibit less misorientation with neighboring grains, resulting in a higher proportion of low-angle grain boundaries (LAGBs) and medium-angle grain boundaries (MAGBs). The lath martensite structure on the surface of the as-received sample, as shown in Figure 3f, has a strong basal texture, and the maximum texture intensity is 10.93.

**Figure 4:**
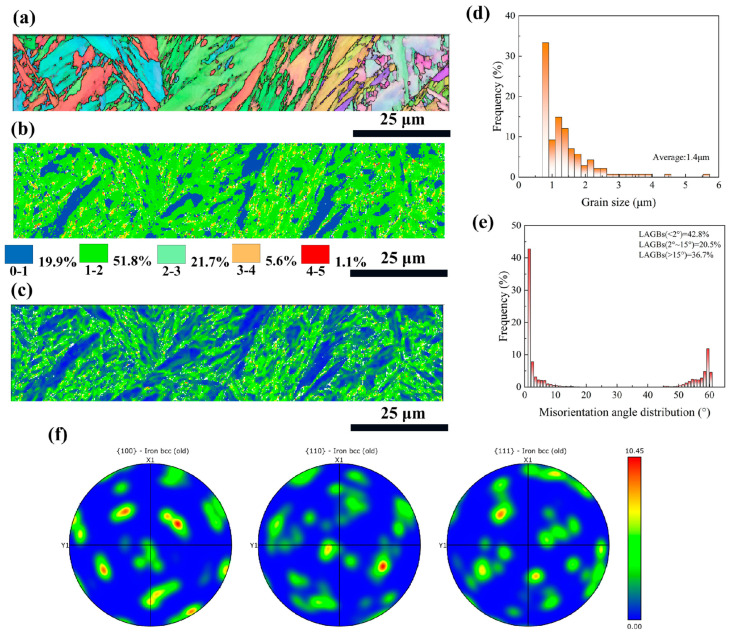
Cross-sectional EBSD results of 75 μJ fs-LSP. (**a**) IPF image; (**b**) KAM image; (**c**) GND image; (**d**) grain size distribution; (**e**) misorientation angle distribution; (**f**) PF image.

After the fs-LSP (75 μJ) treatment, the microstructural characteristics within the near-surface region of 30 μm were characterized, as shown in Figure 4. The laser shock wave induces stress waves on the material surface that exceed the Hugonoit elastic limit, resulting in a severe plastic deformation (SPD) region along the depth direction. Obviously, as shown in Figure 4a–c, equiaxed grains and more small-sized martensitic laths can be identified in the SPD region of the fs-LSP specimen because SPD induced dynamic recrystallization (DRX). The average grain size is only 1.4 μm, representing a 33.3% reduction compared to the as-received sample (Figure 4d). Additionally, SPD led to the dislocation generation and the rearrangement of existing ones, which can be evidenced from the KAM and GND maps. The proportion of KAM values greater than 2 degrees increased from 22.6% in the as-received sample to 28.4% in the fs-LSP sample, and the maximum GND density also increased to 32.3 × 10^14^/m^2^. During the LSP process, localized plastic deformation occurs because grain boundaries obstruct dislocation motion and disrupt plastic flow. When dislocations encounter grain boundaries, they accumulate and form pile-ups, resulting in higher dislocation densities in the areas adjacent to the grain boundaries. Dislocation accumulation and localized plastic deformation further introduced strain and lattice distortion into the material’s microstructure, causing grain boundaries to migrate and potentially creating HAGBs. Figure 4e shows the statistics of the misorientation angles, with the proportion of HAGBs increasing from 25.7% in the as-received sample to 36.7%, consistent with the aforementioned theory and previous studies [27]. Figure 4f shows the texture after fs-LSP, with no significant changes or preferred orientation observed. Only the texture intensity showed a slight decrease, from 10.93 to 10.45, which is known to be attributed to the random rotation of grains after LSP [28].

### 3.3. Wetting Properties

**Figure 5:**
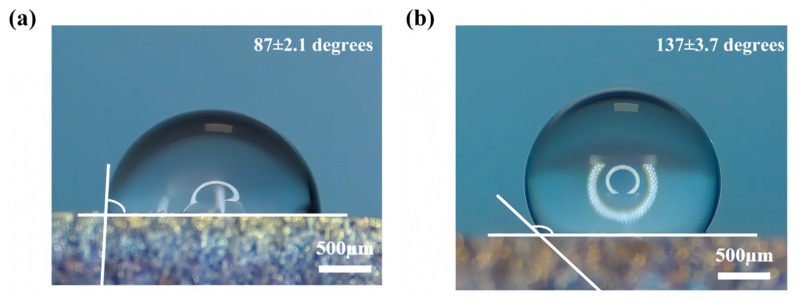
The wettability of the sample (**a**) before and (**b**) after fs-LSP.

Figure 5a,b show the wettability of the sample before and after fs-LSP. The initial sample surface exhibited no texture structure, with a CA of only 87 ± 2.1 degrees, indicating hydrophilicity. In contrast, fs-LSP produced a micro-nano dual-scale structure of LIPSSs and nanoparticles on the target surface, significantly enhancing the material’s hydrophobicity, with the CA increasing to 131 ± 3.7 degrees. The Wenzel and Cassie−Baxter theories are two classical models that define the contact state between a water droplet and material surfaces. The equations for the two states are as follows [29,30]:

(2) $$\cos\theta_{w}=r\cos\theta$$

(3) $$\cos\theta_{CB}=f\left(\cos\theta +1\right)-1$$

where *θ* is the CA on a flat CH1900A surface; r is the surface roughness coefficient; and f is the effective area fraction in contact. The size and morphology of the micro/nano structure determine the roughness or the effective contact coefficient between water droplets and the surface, thereby influencing the final CA. In this regard, the surface structures induced by fs-LSP exhibit significantly higher r and f, which can be considered key factors in the enhancement of hydrophobic performance. Additionally, the storage-induced air pockets in the micro/nano dual-scale hierarchical structure might be another important factor. In previous studies, Barthlott et al. reported that the Salvinia surface, which has an eggbeater-shaped structure, can effectively trap air, thus causing the superhydrophobic structure [31]. Herein, the dual layer-recessed structure formed by ripples and nanoparticles can provide sufficient space to capture and store air. The airborne hydrophobic hydrocarbons also contribute to the final hydrophobicity.

### 3.4. Mechanical Properties

**Figure 6:**
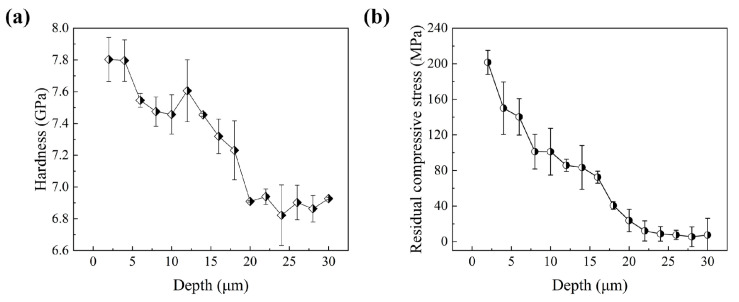
Distribution of (**a**) hardness and (**b**) residual stress along the depth.

Figure 6 shows the results of the nanoindentation test at the interface of the fs-LSP sample, where the maximum hardness reaches 7.8 GPa, which is a 13.2% enhancement with respect to the substrate (~6.9 GPa). The hardness decreases sequentially from the upper surface toward the interior of the material, with the femtosecond laser affecting a depth of ~20 μm, which is similar to previous studies [32]. The hardness enhancement is mainly due to the high density of dislocations and microfine martensitic grains induced by fs-LSP. Under femtosecond laser irradiation, a compressive stress wave is generated at the surface of the material and propagates to the interior, introducing different degrees of plastic deformation in the propagation path and gradually decaying [33,34]. As a result, the hardness tends to decrease until it is the same as that of the substrate. The residual stress shows a similar trend, with the maximum residual compressive stress occurring at the surface, which is about 201 MPa. In general, hardness and residual stress are the key factors for improving wear resistance and fatigue performance, respectively, and thus contribute to the protection of hydrophobic surfaces from damage during service [35].

## 4. Discussion

### 4.1. Effect of Pulse Energy on fs-LSP Results

**Figure 7:**
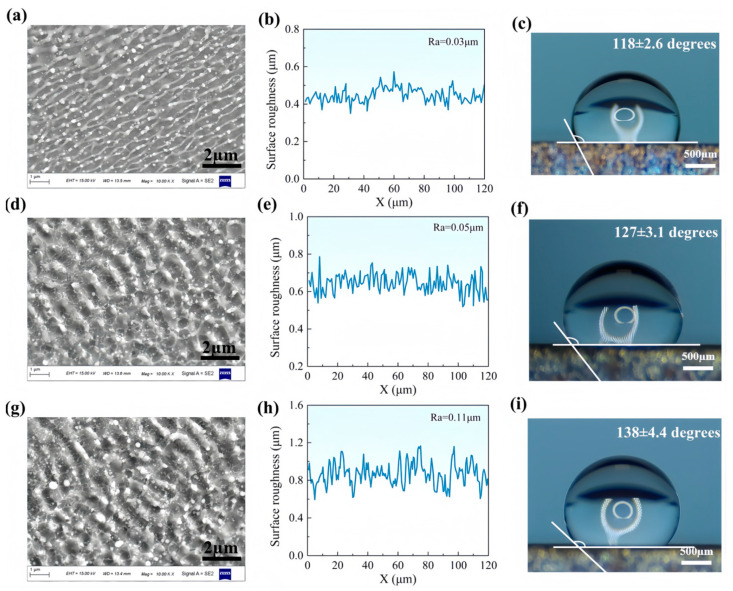
Surface morphology, roughness, and wetting properties after fs-LSP with different pulse energies. (**a**–**c**) 25 μJ; (**d**–**f**) 50 μJ; (**g**–**i**) 100 μJ.

In order to investigate the effect of pulse energy, fs-LSP treatments were conducted using pulse energies of 25 μJ, 50 μJ, and 100 μJ, respectively. The surface topography and roughness of the material are presented in Figure 7. At lower pulse energies, very small-sized LIPSSs were observed to form on the material surface, with a width of approximately 100 nm. A significant number of nanoparticles were found both within the LIPSSs and in the grooves on either side. The overall surface roughness was measured to be 0.03 μm. As the pulse energy was increased, the alignment direction of the LIPSS was observed to deflect by 90 degrees, and the period of the LIPSSs increased to 600 and 900 nm, respectively. Concurrently, the surface roughness was found to rise to 0.07 μm at 50 μJ and 0.11 μm at 100 μJ. Under femtosecond laser irradiation, the energy distribution of subsequent pulses was modulated due to the overlap rate, resulting in a redistribution effect on the LIPSSs. Consequently, the actual energy acting on the material was no longer Gaussian but modulated. It should be noted that fs-LSP involves not only laser-induced stress waves but also intense laser ablation. The protrusions and nanoparticles observed on the surface were primarily attributed to laser ablation and laser melting, which became more pronounced as the laser energy was increased. The increase in the size and roughness of the microstructures was found to provide favorable conditions for the enhancement of the material’s wettability properties. As the pulse energy increases, the contact angle increases from 118 ± 2.6 degrees to 138 ± 4.4 degrees. Obviously, regardless of the energy of fs LSP, the hydrophobic performance is significantly improved compared to the original surface.

**Figure 8:**
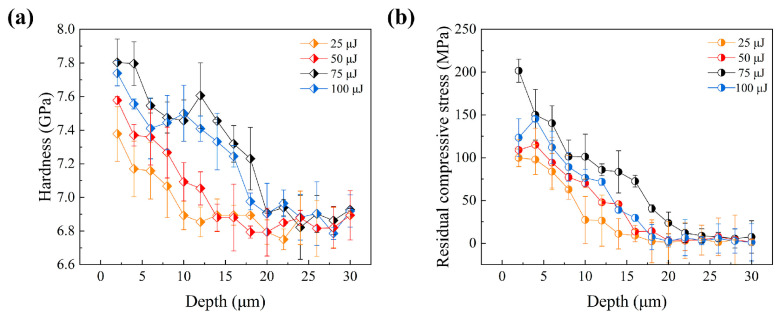
Distribution of (**a**) hardness and (**b**) residual stress after fs-LSP with different pulse energies.

The mechanical properties of the material were also observed to be enhanced with an increase in pulse energy. Figure 8 illustrates the distribution of microhardness and residual stress with depth for different pulse energies, along with their quantitative statistics. It was found that the hardness values, residual stresses, and depth of influence exhibited an increasing trend as the pulse energy was raised. Specifically, the surface hardness increased from 7.38 GPa to a maximum of 7.74 GPa, the maximum residual compressive stress increased from 99 MPa to a peak of 160 MPa, and the depth of influence expanded from 10 μm to a maximum of approximately 18 μm. However, the maximum values for these properties were achieved at a pulse energy of 75 μJ, rather than at the highest tested energy of 100 μJ. In the fs-LSP process, an energy threshold was identified, beyond which excessive energy absorption and concentration at the surface led to processing damage and material melting. Consequently, the strategy of increasing hardness by elevating laser energy was limited by the onset of material damage. Generally speaking, thermal accumulation usually occurs under high repetition-rate laser pulses, which in turn leads to the thermal ablation of the material [36]. Thermal accumulation is closely related to both the pulse interval and the temperature rise caused by each pulse. In this paper, the repetition rate is only 1 kHz; however, when the single pulse energy is large enough, the temperature rise it causes is difficult to dissipate completely and will still remain until the arrival of the next pulse. Therefore, the thermal accumulation may still exist. The variations in hardness and residual stress were also noted to contribute to differences in wear and fatigue performance, highlighting the complex interplay between laser parameters and material response [37].

**Figure 9:**
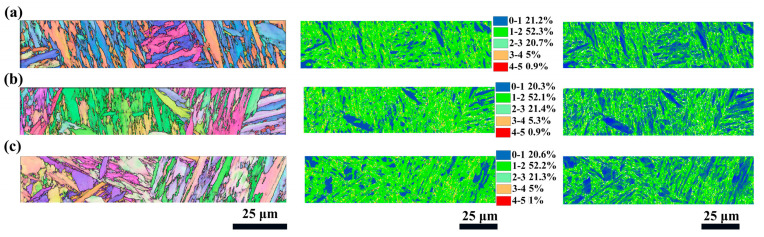
EBSD results after fs-LSP with different pulse energies. (**a**) 25 μJ; (**b**) 50 μJ; (**c**) 100 μJ.

**Figure 10:**
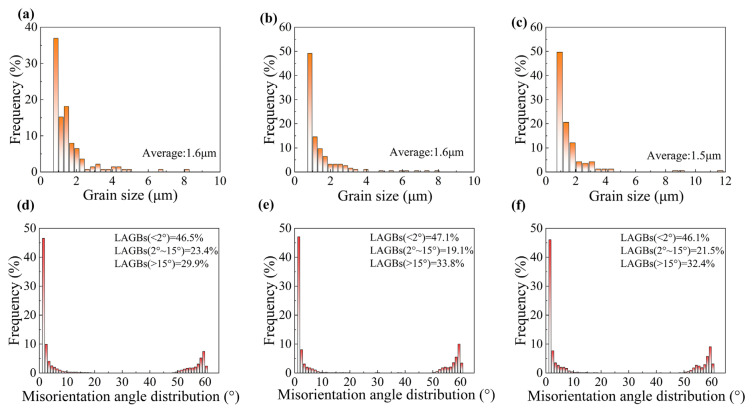
Grain size and misorientation angle distribution after fs-LSP with different pulse energies. (**a**,**d**) 25 μJ; (**b**,**e**) 50 μJ; (**c**,**f**) 100 μJ.

The enhancement of mechanical properties was closely associated with the microstructural evolution of the material induced by fs-LSP, as depicted in Figure 9. KAM analysis revealed that as the pulse energy increased, the proportion of regions with KAM values exceeding 2° was measured at 26.6%, 27.6%, and 27.3%. GND density analysis indicated that the maximum GND densities were observed to be 31.3 × 10^14^/m^2^, 31.9 × 10^14^/m^2^, and 32 × 10^14^/m^2^. Notably, EBSD analysis demonstrated that the laser pulse energy did not significantly alter the grain size, as presented in Figure 10a–c. Furthermore, the orientation difference angle statistics revealed that the percentage of HAGBs exhibited an increasing trend with higher pulse energies, with values of 53.3%, 52.9%, and 53.9%, as shown in Figure 10d–f. However, when the pulse energy reached 100 μJ, a reduction in the material strengthening effect was observed. This decline was primarily attributed to the absence of an absorption layer during fs-LSP, where the laser energy was directly applied to the material surface. The excessive laser energy led to an increase in surface roughness, which negatively impacted the material’s ability to absorb laser energy efficiently [20]. Laser ablation under single pulses and thermal accumulation between pulses have exacerbated the occurrence of the above-mentioned phenomenon. On the one hand, compared with low energy pulses, more severe ablation occurs on the surface under a single pulse energy of 100 μJ, including the formation of larger sized LIPSSs. On the other hand, the thermal accumulation between pulses is intensified due to the increased temperature rise caused by a single pulse, which further softens the surface microstructure of the material. This phenomenon further underscores the existence of an optimal laser energy threshold for achieving maximum mechanical property enhancement.

### 4.2. Effect of the Number of Impacts on fs-LSP Results

**Figure 11:**
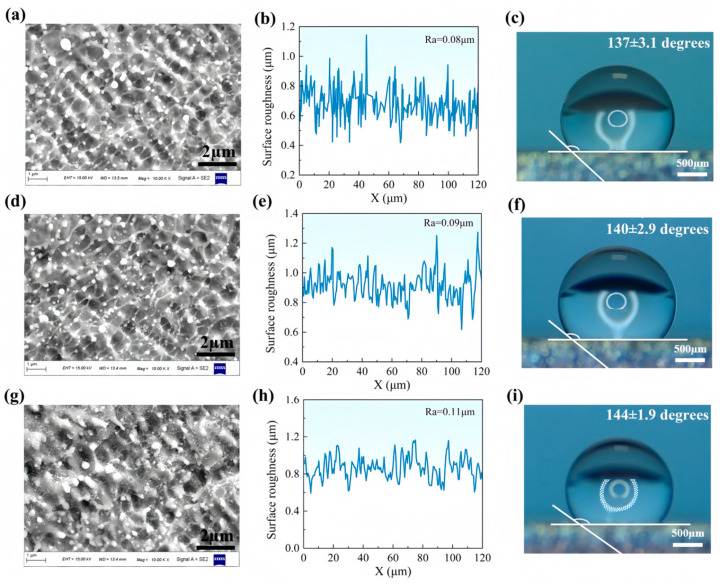
Surface morphology, roughness, and wetting properties after fs-LSP with different number of impacts. (**a**–**c**) N = 3; (**d**–**f**) N = 6; (**g**–**i**) N = 9.

Fs-LSP operates on the material surface through high energy-density laser pulses applied within an extremely short time frame, generating intense shock waves and thermal effects. This process induces plastic deformation and microstructural changes on the material surface. As shown in Figure 11, experiments were conducted with impact times of 3 (N = 3), 6 (N = 6), and 9 (N = 9), respectively. All the experiments with different impact times were carried out at a pulse energy of 75 μJ. The effect of impact times on the LIPSS period is not significant, and the periods under different impact times are 800 nm (N = 3), 850 nm (N = 6), and 850 nm (N = 9). Additionally, the results showed that with the increase in impact times, surface roughness increased and the LIPSS gradually degraded. When N = 9, the surface roughness Ra increases from 0.08 μm to 0.11 μm. The accumulation of thermal effects leads to melting, recasting, or oxidation of the material surface, which disrupts the original LIPSS [38]. In addition, due to the plastic deformation caused by multiple shocks, the surface microstructure becomes more complex, which further intensifies the increase in surface roughness and reduces the absorption rate of laser energy. For wettability performance, the surface exhibited substantial contact angles after all impact times, specifically at 137 ± 3.1, 140 ± 2.9, and 144 ± 1.9 degrees. Similar to the results of pulse energy, the increase in roughness and surface nanoparticles is the main reason for the increase in contact angle.

**Figure 12:**
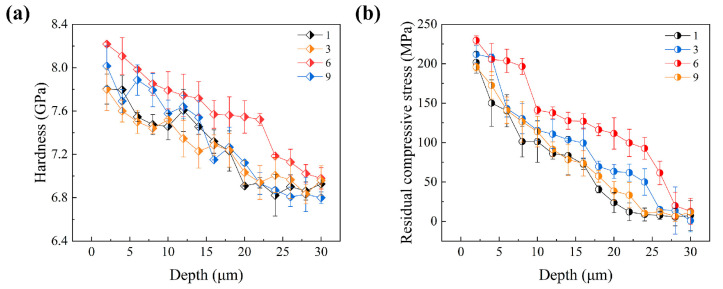
Distribution of (**a**) hardness and (**b**) residual stress after fs-LSP with different number of impacts.

With the increase in impact times, the microhardness increases continuously, reaching a maximum value at N = 6 (8.2 GPa), as shown in Figure 12a. When the residual stress is N = 6, the impact depth reaches a maximum of 30 μm, but with the increase in impact times to N = 9, the surface residual stress decreases, as shown in Figure 12b. During the multiple laser shock processes, each shock acts on the surface that has been affected by the previous one, which may have experienced remelting or have relatively high roughness. Different from the original smooth solid-state surface, after absorbing laser energy, such a surface introduces stress waves into the material while softening itself. Under the combined action of these two factors, the final strengthening result is closely related to the number of shocks. An excessive number of impacts will introduce a strong softening effect, which in turn reduces the effectiveness of fs-LSP. During the fs-LSP process, the laser pulse acts on the material surface within an extremely short time, generating high-intensity shock waves and thermal effects. The plastic deformation induced by the shock wave introduces residual compressive stresses on the surface and subsurface of the material, thereby enhancing its mechanical properties. However, as the number of impacts is increased, the accumulation of thermal effects causes localized temperature rises in the material. This may trigger stress relaxation or microstructural changes, which weaken the depth of influence of the residual stresses. When the cumulative temperature change from the thermal effects exceeds the residual stress introduced by the laser-induced plastic deformation, the impact depth is reduced. Nevertheless, the residual stresses introduced remain larger and deeper compared to those in the original material.

**Figure 13:**
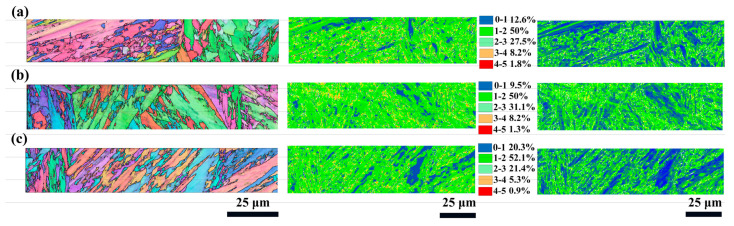
EBSD results after fs-LSP with different number of impacts. (**a**) N = 3; (**b**) N = 6; (**c**) N = 9.

**Figure 14:**
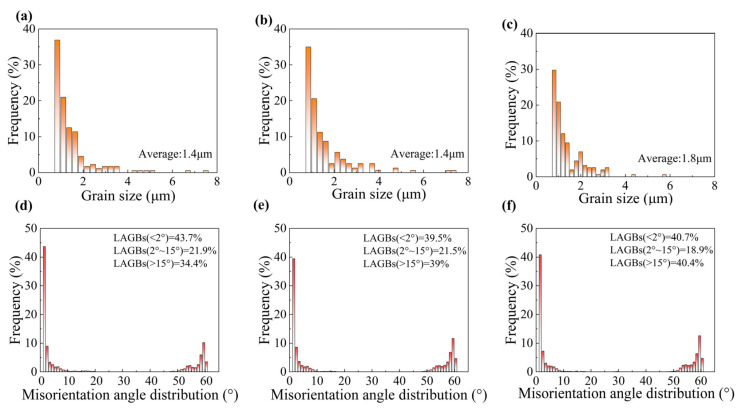
Grain size and misorientation angle distribution after fs-LSP with different number of impacts. (**a**,**d**) N = 3; (**b**,**e**) N = 6; (**c**,**f**) N = 9.

In order to investigate the intrinsic mechanism of the shock times on fs-LSP, the material tissue characteristics at three different pulse energies of 3, 6, and 9 times were systematically characterized in this study, as shown in Figure 13. The KAM analysis shows that with the increase in the number of impacts, the percentage of regions with KAM values larger than 2° is 37.5%, 40.6%, and 32.9%, which indicates that the dislocation density and the localized strain inside the material reach the peak at N = 6. The average grain size inside the material gradually increases with the increase in impact times, reaching a maximum value of 1.8 μm when N = 9, as shown in Figure 14a–c. In addition, the orientation difference angle statistics showed that the proportion of HAGB showed an increasing trend with the increase in energy, which were 56.3%, 60.5%, and 59.3%, as shown in Figure 14d–f, and the increase in the proportion of HAGB indicated the enhancement of the strengthening effect of grain boundaries. With the increase in the number of impacts, the accumulation of thermal effects may lead to an increase in the local temperature, which activates the dislocation motion and promotes stress relaxation, whereas the increase in the surface roughness may reduce the effective absorption of laser energy and affect the uniformity of the strengthening effect. These factors work together to limit further optimization of the microstructure.

## 5. Conclusions

In this paper, a fs-LSP method is proposed and successfully applied to manufacture surfaces with both hydrophobic and mechanical properties. The optimization effect of femtosecond laser parameters on the surface morphology, microstructure, and mechanical properties of the material was systematically analyzed by modulating the pulse energy and the number of impacts. The main conclusions are as follows:

(1) The fs-LSP method achieved a comprehensive improvement in both the hydrophobicity and mechanical properties of the CH1900 steel surface. The refinement of surface grains, increase in dislocation density, and other microstructural transformations are the reasons for the enhancement of mechanical properties, while the hydrophobicity originates from the LIPSS. When the pulse energy is 75 μJ and the number of impacts is 6, the surface hardness reaches maximum (8.2 GPa), and the contact angle reaches 140 degrees.
(2) With the increase in pulse energy, the LIPSS size increases, the alignment direction changes, and surface roughness increases, collectively creating favorable conditions for the improvement in material wettability. At the same time, the microhardness and residual stress increase significantly, reaching the maximum when the pulse energy is 75 μJ. However, high energy will cause surface damage and material melting, which will weaken the strengthening effect, which indicates that the optimization of pulse energy plays a key role in achieving the best strengthening effect of fs-LSP.
(3) Excessive impact times lead to the destruction of the LIPSS and the increase in the average grain size, which is due to the accumulation of thermal effects leading to melting, oxidation, and stress relaxation, which limits the further optimization of the microstructure. It indicates that the number of impacts needs to be optimized to achieve the best strengthening effect. In the current study, the optimal surface strengthening effect can be achieved with 6 impact times.
(4) The proposed fs-LSP technology provides a strategy for the fabrication of hydrophobic surfaces with excellent mechanical properties. Additionally, fs-LSP technology is expected to further optimize the multifunctional performance of materials and promote its application in high-end fields such as aerospace.

## Data Availability

The original contributions presented in this study are included in the article. Further inquiries can be directed to the corresponding author.
